# Optoelectrical Properties of Treated CdSe Thin Films with Variations in Indium Chloride Concentration

**DOI:** 10.3390/ma16114108

**Published:** 2023-05-31

**Authors:** Hasrul Nisham Rosly, Camellia Doroody, Muhammad Najib Harif, Ili Salwani Mohamad, Mustapha Isah, Nowshad Amin

**Affiliations:** 1 College of Engineering, Universiti Tenaga Nasional (The Energy University), Jalan IKRAM-UNITEN, Kajang 43000, Malaysia; 2 Faculty of Electrical and Electronic Engineering Technology, Universiti Teknikal Malaysia Melaka, Durian Tunggal, Melaka 76100, Malaysia; 3 Institute of Sustainable Energy, Universiti Tenaga Nasional (The Energy University), Jalan IKRAM-UNITEN, Kajang 43000, Malaysia; 4 Faculty of Applied Sciences, Universiti Teknologi MARA, Cawangan Negeri Sembilan, Kuala Pilah, Seremban 72000, Malaysia; 5 Faculty of Electronic Engineering Technology, Universiti Malaysia Perlis, Arau 02600, Malaysia; 6 Department of Physics, Kaduna State University, Kaduna 2339, Nigeria

**Keywords:** solar cells, cadmium selenide, indium chloride, thin film, chloride treatment, magnetron sputtering, energy

## Abstract

The effect of a nontoxic chloride treatment on the crystallinity and optoelectrical characteristics of a CdSe thin film was studied. A detailed comparative analysis was conducted utilizing four molarities (0.01 M, 0.10 M, 0.15 M, and 0.20 M) of indium (III) chloride (InCl_3_), where the results showed a notable improvement in CdSe properties. The crystallite size of treated CdSe samples increased from 31.845 nm to 38.819 nm, and the strain in treated films dropped from 4.9 × 10^−3^ to 4.0 × 10^−3^, according to XRD measurements. The highest crystallinity resulted from the 0.10 M InCl_3_-treated CdSe films. The In contents in the prepared samples were verified by compositional analysis, and FESEM images from treated CdSe thin films demonstrated compact and optimal grain arrangements with passivated grain boundaries, which are required for the development of a robust operational solar cell. The UV-Vis plot, similarly, showed that the samples were darkened after treatment and the band gap of 1.7 eV for the as-grown samples fell to roughly 1.5 eV. Furthermore, the Hall effect results suggested that the carrier concentration increased by one order of magnitude for samples treated with 0.10 M of InCl_3_, but the resistivity remained in the order of 10^3^ ohm/cm^2^, suggesting that the indium treatment had no considerable effect on resistivity. Hence, despite the deficit in the optical results, samples treated at 0.10 M InCl_3_ showed promising characteristics as well as the viability of treatment with 0.10 M InCl_3_ as an alternative to standard CdCl_2_ treatment.

## 1. Introduction

Solar cells are drawing great attention with the increase in world energy consumption, for solar energy is well identified as the most cost effective and cleanest resource [1]. As well as the common Si-based solar cells, thin films made by depositing various semiconductor thin films have been developed as second-generation solar cells with an increasing market share and competitive cost [2]. Semiconductors are critical elements in thin film configurations, and cadmium selenide (CdSe) is a group II-VI semiconductor, well regarded as a photoactive substance owing to its high absorption coefficient of 10^5^ cm^−1^ and optimal band gap of 1.74 eV [3]. Using CdSe in solar cells with an optimal thickness and carrier concentration of 10^16^ to 10^18^ cm^−3^ as a window layer has been a breakthrough, resulting in a major achievement of Jsc improvement and 22.1% CdTe thin film efficiency in 2016 [4]. Several techniques are available to deposit thin CdSe films such as RF magnetron sputtering [5], chemical bath deposition (CBD) [6], thermal evaporation [7], and quantum dots [8], depending on the desired specifications in the produced thin film [9]. The RF sputtering process is renowned for its ability to provide uniform film deposition without ion damage over a large area [10,11]. Moreover, the performance of CdSe thin films can be optimized by altering the properties concerned for an absorber or window layer using chloride treatment [12]. Chloride treatment is widely established as a spectacular step, which can be performed by a wet or dry process, using chloride compounds such as CdCl_2_, MgCl_2_, and InCl_3_ [13,14]. CdCl_2_, however, is very toxic, posing a threat to industrial and environmental cycles. Furthermore, despite its practicality, CdCl_2_ treatment imposes high unavoidable production cost [15]. There have recently been efforts to introduce alternative treatments for Cd-based thin films that have yet to be found effective [16]. These are potential salts including XCl_2_, where X stands for different metal ions with the same anion as InCl_3_, identified in other works to improve the electrical properties of thin films [17,18,19]; however, the carrier concentration and Voc improvement after the treatment was demonstrated to be much smaller compared to conventional CdCl_2_ in most of the cases, except for InCl_3_-treated samples [20,21,22]. Indium in a solid compound was found to generate a type of point defect that functioned as ions in a dilute solution of a strong electrolyte [23]. In addition, other reports have indicated that InCl_3′_s lack of direct metal–ligand bonds improves hydrolysis, consuming less reaction energy as a treatment solution [24]. Accordingly, the In^3+^ ions occupy the Cd^2+^ site in the CdSe lattice and free Cl^−^ ions effectively form bonds with the In^3+^ and Cd^2+^ surface atoms, remarkably preventing the Ostwald ripening occurrence and allowing for size control of nanoparticles, resulting in a more uniform surface and grain structure when using InCl_3_ [25]. However, a full understanding of the treatment process mechanism is required [26]. In this regard, this study is conducted to investigate the structural and optoelectrical effects of InCl_3_ (0.01 M, 0.10 M, 0.15 M, and 0.20 M) molar concentration on CdSe thin films. The primary goal of this study is to identify the optimal molar concentration of InCl_3_ as a feasible and environmentally friendly compound to be used for solar cell applications.

## 2. Experimental Details

The experimental procedure is classified to two main steps, including the deposition of CdSe using an RF magnetron sputtering method, and the treatment of prepared CdSe samples by indium chloride at various molarities followed by the thermal annealing.

### 2.1. CdSe Thin Film Growth

**Figure 1:**
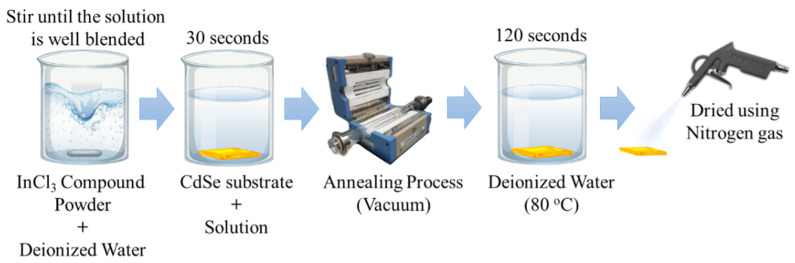
Indium chloride treatment process.

Borosilicate glass (3 cm x 3 cm x 1.1 mm) was used as the substrate in this study. Firstly, the glass substrates were ultrasonicated in acetone, methanol, and deionized water. CdSe films with a thickness of approximately 100 nm were grown by sputtering at room temperature. Sputtering deposition of thin films typically involves reactive gases, in this case, argon (Ar), in the chamber and energetic electric cathodes that produce self-sustaining plasma. The CdSe 5 cm in diameter target with the purity of 99.95% was purchased from Matsurf Technologies Inc. The sputtering chamber vacuumed to a base pressure of 1.5 × 10^−5^ Torr via both and film growth was conducted for 15 min with 40 Watt power, 5 SCCM argon flow, and 2.0 × 10^−2^ Torr processing pressure. When the deposition process was performed, the deposition pressure reading was taken at an average reading of 2.0 × 10^−2^ Torr. Ar gas was used as an ambient due to its low reactivity and high sputter yield. A Kurt J. Lesker radio frequency (RF) magnetron sputtering, equipped with an in situ substrate holder, four quartz halogen lamp heaters, and a sputter down distance of 14 cm were utilized to deposit 100 nm CdSe thin film. All sputter depositions were carried out at room temperature. Prior to the sputtering process, substrates were inserted in the chamber and one hour pre-heating at a set temperature was applied to eliminate any residues and prevent nonuniform heating during the deposition process. After the CdSe deposition, thin films went through the indium chloride treatment process. Kanto Chemical Co., Inc., Tokyo, Japan. supplied pure 3N5 indium chloride (99.95%), which was used without further purification. Figure 1 shows the step-by-step chloride treatment process carried out in this study.

The solutions with different indium chloride concentrations of 0.01 M, 0.10 M, 0.15 M, and 0.20 M were prepared by dissolving indium chloride compound powder into deionized water for 30 min using magnetic stirrer. Afterwards, CdSe surface was treated by immersing the CdSe substrates into the solutions for 30 s. The wet substrates were then immediately dried in open air under the fume hood. The dried substrate was then immediately loaded into the thermal annealing chamber. After the annealing furnace was evacuated to 50 × 10^−3^ Torr, with a continual temperature increase of 25 °C/min, the interior tube reached 400 °C. The heat treatment step was completed within 10 min in a vacuum pressure of 100 to 150 m Torr.

### 2.2. CdSe Thin Film Characterization

The structural properties and phases were inspected at room temperature by X-ray diffraction using X-ray diffractometer-6000 (Shimadzu, Kyoto, Japan) and Cu Kα radiation to record a 2 theta range from 20° to 80° with a step size of 0.01°. Veeco Dektak 150 profilometer (Oyster Bay, NY, USA) was used to evaluate the film thickness. The morphology images and grain size were obtained with a Carl Zeiss Merlin field emission scanning electron microscopy (FESEM Carl Zeiss, Oberkochen, Germany) at an accelerating voltage of 15 kV. The compositions of the films were traced by energy dispersive spectroscopy (EDX) using multi-dimensional Helios system, and optical analysis was carried out using a Lambda-900 UV-Vis spectrophotometer (PerkinElmer, Waltham, MA, USA) in the range of 200-1000 nm. The electrical characteristics were measured using an ECOPIA HMS-3000 (ECOPIA, Anyang, Korea) Hall effect device with the probe current and magnetic field of 45 nA and 0.57, respectively.

## 3. Results and Discussions

### 3.1. Structural Properties Analysis

**Figure 2:**
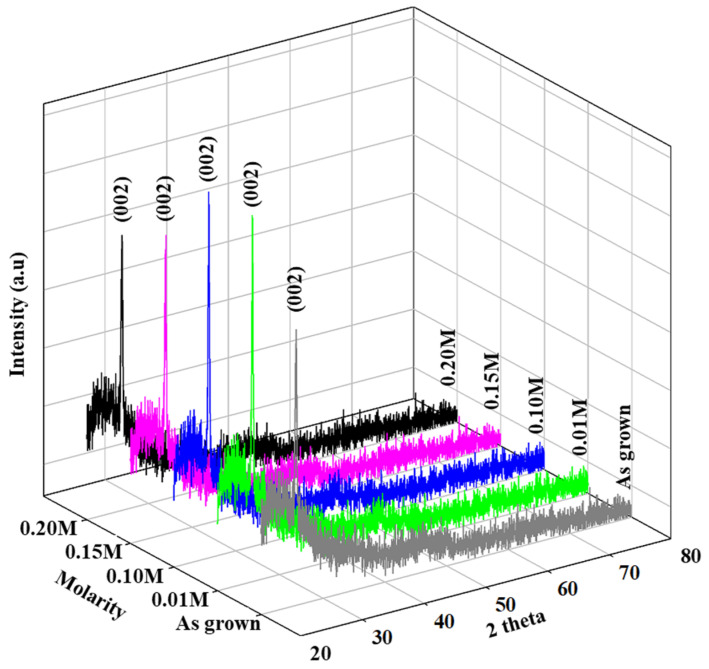
X-ray diffractograms of CdSe treated by different InCl_3_ molarity.

The X-ray graphs of as-grown and InCl_3_-treated thin CdSe films are plotted in Figure 2 showing the (002) plane with hexagonal orientation at 2 theta = 24.12° for all the samples also validated by JCPDS data card No. 01-075-5680 [27]. It was observed that the preferred CdSe (002) plane turned out to be more intense in all InCl_3_-treated thin films compared to the as-deposited CdSe, which indicates the optimized crystallinity, passivated boundaries, and grain refinement in treated thin films [28]. Aside from the major peak, the other slopes were small attributed to the increase in preferred crystallite orientation in the indium-treated thin films. In Figure 2, the addition of In^3+^ ions bonding with Cd^2+^ and free Cl^−^ in CdSe especially on indium treatment at 0.01 M and 0.10 M was found to increase the peak intensity or the optimal crystallinity. It was observed that an indium concentration of 0.10 M resulted in a favorable structural property validated by the intensive diffraction peak shown in Figure 2. However, for heavily indium-doped CdSe such as indium treatment at 0.15 M and 0.20 M, the strength of the CdSe peak drops and peaks broadened, showing that local defects formed in the excessively doped CdSe lattice [29], which also corresponded to a decrease in the average crystal size, D, of the films [30].

Various structural evaluations were performed utilizing relative equations in order to derive the precise structural specifications [31] and are presented in Table 1. The lattice parameters were estimated to be in the range 4.238–4.254 Å for a and 6.920–6.747 Å for c in accordance with the previous studies [32,33]. This indicates that the CdSe lattice was not dramatically affected by the atomic density of indium [30]. From Table 1, the crystallite size was found to increase after the indium treatment was performed. The crystallite size for the as-grown without treatment sample was in the average 31.845 nm, while the crystallite size for the sample that underwent indium treatment was in the range 37.223–38.819 nm where indium treatment at 0.10 M had the largest crystallite size, which was 38.819 nm, indicating an improvement above the crystallite size reported in prior studies utilizing CdCl_2_ treatment [28,34]. This exemplifies that indium treatment has the potential to increase the size and grain size of CdSe crystallites, implying improved device performance and lifetime [35]. 

**Figure 3:**
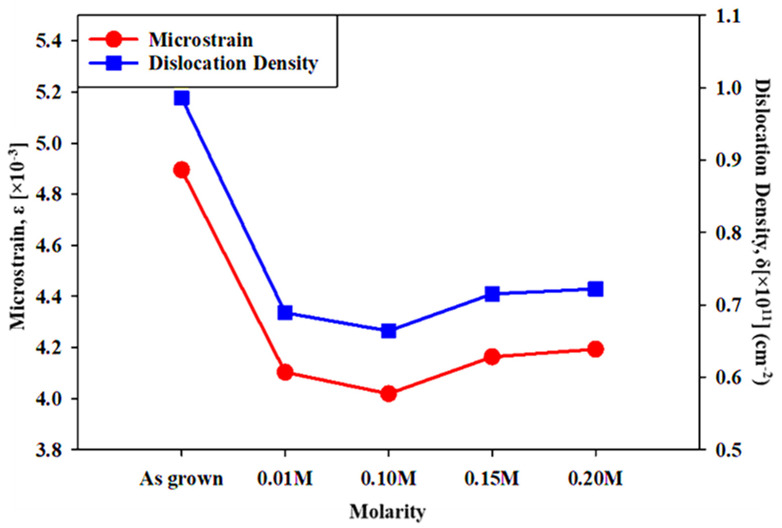
Dislocation density and microstrain of CdSe treated by different InCl_3_ molarities.

The indium treatment strongly influences the dislocation density and microstrain of CdSe films. Figure 3 shows the changes in the dislocation density and microstrain for various indium treatment molarities. It is confirmed from Figure 3 that dislocation density decreased when adding the indium molar concentrations of 0.01 M and 0.10 M, thereafter a slight increase was observed. Microstrain also showed a similar trend where there was a decrease in microstrain with an increase in indium concentration up to 0.10 M and a slight increase thereafter. The lowest dislocation density and microstrain value were obtained in the indium treatment at 0.10 M, which brought values of 0.664 × 10^11^ cm^−2^ and 4.019 × 10^−3^, respectively. Incorporation of the In:CdSe thin films with the reduced microstrain and fluctuation in interplanar spacing, and the overall altered characteristics may result in a higher solar cell performance. Studies on the functional dependency of strain and dislocation density with indium concentration indicate that the strain and dislocation density decrease with indium concentration, whereas the crystallite size increases [36].

### 3.2. Morphology and Compositional Analysis

**Figure 4:**
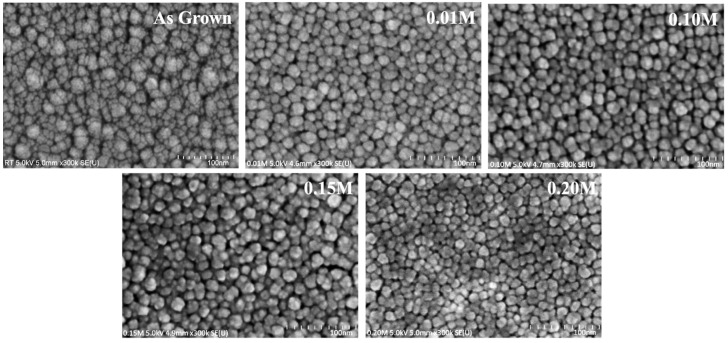
FESEM image of CdSe treated by different InCl_3_ molarities.

The grain structure of the as-grown and InCl_3_-treated CdSe thin films was screened using FESEM images. As shown in Figure 4, the surface morphology was more uniform, pin hole free, and compact without cracks in the treated samples. XRD outputs confirmed that the increase in crystallinity caused by post-heat treatment changed the surface morphology of CdSe samples [20]. The grain size was within the range of 30–42 nm, which could be compared with the film thickness and is well supported by XRD results demonstrated in an earlier report [5].

**Figure 5:**
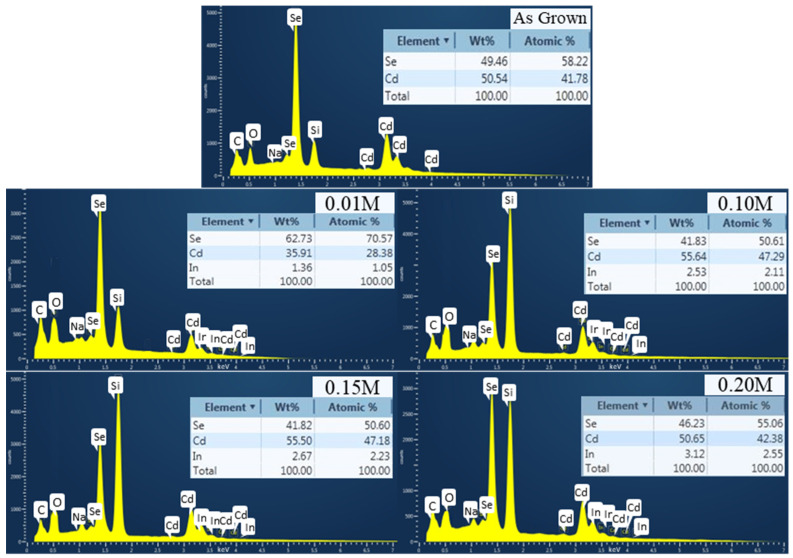
EDX patterns of as-grown and InCl_3_-treated CdSe thin films.

However, there was no significant change in grain size between the as-grown and treated films estimated through FESEM. The sizes of the crystallite as-grown films were found to be slightly smaller than those of treated films, which was also evidenced by XRD results. The heat treatment required to relocate grain boundaries resulted in the coalescence of adjacent grains by eliminating their shared grain boundaries and the substantial grain expansion of InCl_3_-treated films annealed at 400 °C [9]. Moreover, the incorporation of In into thin CdSe films decreases microstrain and fluctuations in interplanar spacing, resulting in improved thin film properties. As a result, it is critical to examine the content of In in CdSe thin films. Here the EDX spectra presented in Figure 5 reveal a modest rise in the In atomic ratio with increasing dopant molarity in line with previous studies [37]. The presence of other peaks such as Si, Na, C, and O represents the glass composition that was used as the substrate material.

Furthermore, thermal annealing provides sufficient energy to break irregular connections within the atom structure of the as-grown CdSe film [31]. The primary force generated by the rearranged bonds between the indium dopants and CdSe atoms enhances the overall atomic orientation, and this can lead to the formation of optimal CdSe crystals as presented here in the XRD and EDX results [38]. Chlorine treatment at higher temperatures may contribute to enhanced grain configuration and passivated grain borders, where both of these features are necessary for highly stable operational solar cells [39].

### 3.3. Optical Analysis

**Figure 6:**
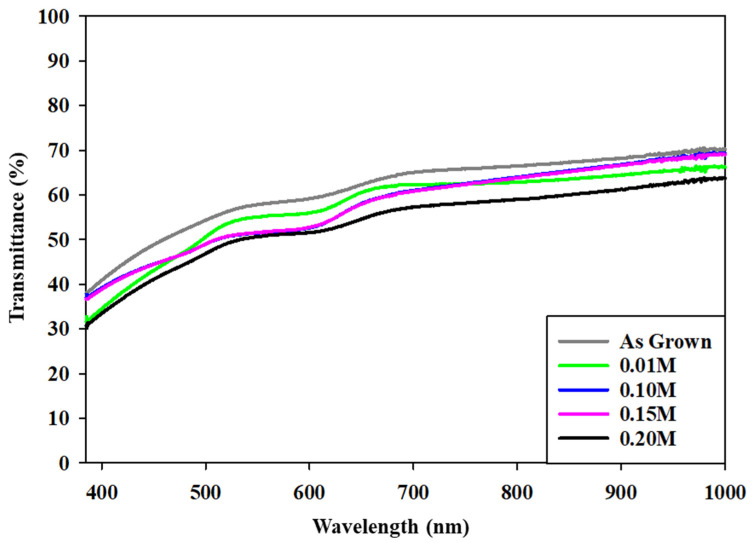
Transmittance spectra for the as-grown and InCl_3_ treatment of CdSe thin films.

CdSe thin films were examined regarding transmittance profiles as plotted in Figure 6, within wavelengths of 385–1000 nm for analyzing the optical characteristics of the based and InCl_3_-treated CdSe films. Transparency was substantial for all samples in the near-infrared (NIR) range, and it fell drastically for the reduced visible-wavelength spectra. This reduction can be explained as the photon energy in the near-infrared band being under the prohibited band energy; electrons in CdSe films cannot be excited from the valence to the conduction band. In other words, this declination is due to the poor energy absorption in this area. 

**Figure 7:**
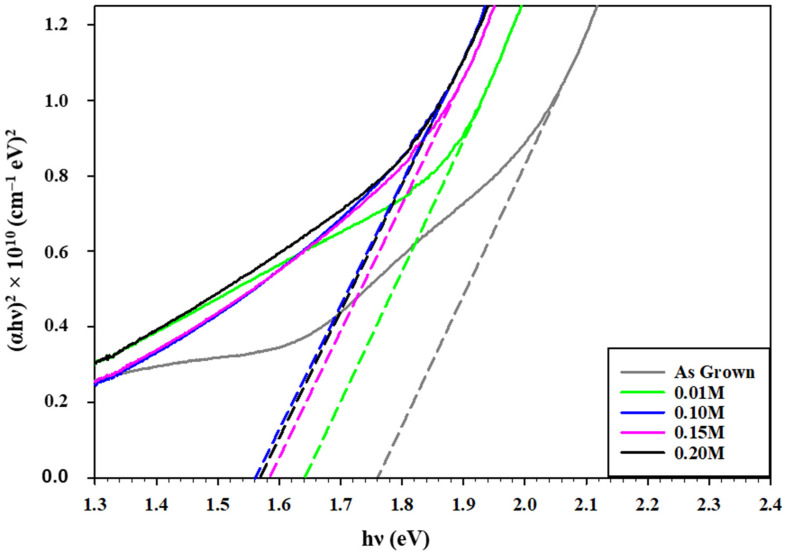
Tauc plot for the as-grown and treated CdSe by different InCl_3_ molarities.

Visible photons have more energy than NIR incident light, which can be absorbed by excited electrons and other trap levels that may be present throughout the band gap [38]. Eventually, an increased doping level of the indium solution can cause the deteriorated transmission due to the inserted indium dopants into the trap levels within forbidden bands [40]. The reduced visible transmittance of the treated CdSe films is a reliable indicator of CdSe’s suitability to be used as an absorber layer in solar cells’ configuration. The wavelength range from 200 to 1000 nm was depicted to analyze the optical absorption of the prepared samples. Here, the band gap range and transition mechanism were determined by analyzing the optical density deviations with wavelength. A plot of (αhν)^2^ against hν was projected from the estimated value of the absorption coefficients toward the break point of α = 0. Tauc’s plots on the CdSe as-grown and treated samples with InCl_3_ are presented in Figure 7, visualizing the band gap of the as-grown CdSe to be 1.76 eV, which is also tabulated in Table 2. The optical band gap decreased by up to 1.56 eV when InCl_3_ treatment was performed. Indium defect states originating in the forbidden gap may cause the absorption of incoming photons and a substitutional dissolution, resulting in an improvement in the grain structure of the film as well as the reduction in the band gap value [41]. 

The results in Figure 7 are consistent with earlier research that showed a smaller band gap following heat treatment and CdSe’s potential as an absorber layer [42]. The reduced band gap could happen owing to an increase in lattice rearrangements caused by the entry of dopants in the host crystal structure [43]. Ultimately, it was demonstrated that indium doping can boost CdSe’s quality as an absorber in solar cell configuration by narrowing the optical energy band gap. The optical study in this paper shows that utilizing InCl_3_ reduces the band gap compared to using CdCl_2_, as shown in previous studies, which reported an increase in band energy after treatment [34].

### 3.4. Electrical Properties’ Analysis

**Figure 8:**
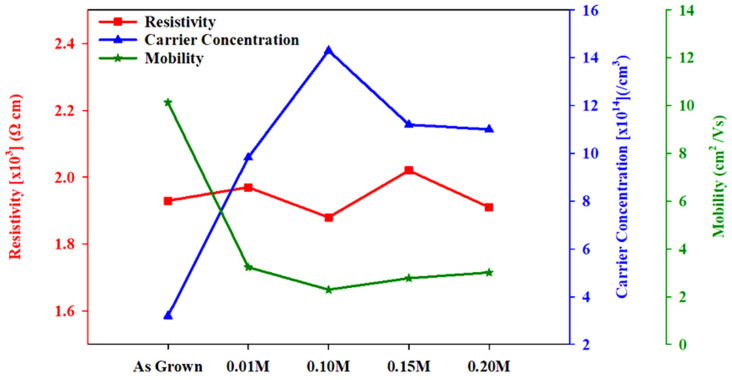
Electrical properties of as-grown and treated CdSe thin films.

To investigate the electrical properties of the film, the Hall effect was measured. CdSe has abundant selenium vacancies and is an n-type conductor by nature. If the doping or treatment results in more cadmium vacancies in the films, the conductivity might shift to p-type [44]. From the measurement tabulated in Table 3, all films were proven to be n-type semiconductors. From this, it is clear that the doped indium acts as the donor in CdSe films. Figure 8 shows the carrier concentration, resistivity, and mobility variation with different indium molarity treatments. The carrier concentration first increased with increasing indium molarity and reached a maximum point. This maximum point was at 0.10 M indium treatment and carried a value of 1.43 × 10^15^ cm^−3^. As reported before, indium can enter into Cd sites of CdSe crystals substitutionally and can act as donors [45]. 

The increase in the carrier concentration of a film at the rather low concentration indium treatment stages (0.01 M and 0.10 M) is thought to be due to this effect. However, the carrier concentration decreased at 0.15 M and 0.20 M treatment. Hayashi et al. stated that the radius of an indium ion is known to be smaller than the Cd ion. Therefore, a CdSe lattice doped with substitutional indium atoms may shrink and become distorted. The distorted lattice reduces the donor action and the carrier concentration becomes small [46]. Figure 8 also shows the mobility on the as-grown CdSe thin film was 10.13 cm^2^/volt-sec and exhibited a significant decrease when indium treatment was performed. However, mobility for all indium molarities showed a similar range in value that was between 2.29–3.23 cm^2^/volt-sec. These results reveal that impurity scattering limits the mobilities [47]. 

The resistivity was found in the order of 10^3^ Ω cm, indicating no significant effect on resistivity by the indium treatment performed. Despite that, the lowest resistivity of 1.88 × 10^3^ Ω cm was found for the indium treatment at 0.10 M. This limited resistance is probably due to the higher carrier concentration.

## 4. Conclusions

To verify the feasibility of nontoxic chloride treatment on CdSe thin films, the study on the effectiveness of four different molarities of a nontoxic treatment compound of InCl_3_ on CdSe thin films was initiated. The structural studies revealed the preferential orientation of (002) with hexagonal phase for the as-deposited and treated films, and the highest peak intensity was found for the samples treated at 0.10 M-concentrated InCl_3_. The incorporation of In and its compositional ratio was confirmed by EDX analysis. The observed grain size and direct band gap values for the as-grown and treated samples were found to be in the range of 31–42 nm and 1.7 to 1.5 eV, respectively. The treated samples exhibited one order increase in carrier concentration with the optimal value of 1.43 × 10^15^ /cm^3^ obtained from the 0.10 M InCl_3_-treated CdSe thin films. The results indicate that CdSe with 0.10 M InCl_3_ treatment provides the optimum value as a prospective n-type semiconductor for solar cell applications due to its structure, grain growth, high carrier concentration, and low resistivity, but not for its optical band gap suitability. Therefore, all the findings support the use of environmentally friendly InCl_3_ treatments in place of traditional CdCl_2_, to reduce ecological impacts and manufacturing costs.

## Figures and Tables

**Table 1:** Structural factors of CdSe treated by different InCl_3_ molarity.

Molarity	hkl	a (Å)	c (Å)	D (nm)	ε (×10^−3^)	δ (×10^11^) (cm^−2^)
As-grown	(002)	4.243	6.928	31.845	4.895	0.986
0.01 M	(002)	4.254	6.947	38.090	4.103	0.689
0.10 M	(002)	4.246	6.934	38.819	4.019	0.664
0.15 M	(002)	4.238	6.920	37.399	4.163	0.715
0.20 M	(002)	4.248	6.937	37.223	4.193	0.722

**Table 2:** Energy band gap of CdSe treated by different InCl_3_ molarities.

Molarity	Energy Band Gap (E_g_)
As-grown	1.76
0.01 M	1.64
0.10 M	1.56
0.15 M	1.57
0.20 M	1.58

**Table 3:** Electrical parameters of as-grown and treated CdSe by different InCl_3_ molarities.

Molarity	Carrier Concentration (/cm^3^)	Mobility (cm^2^ /Vs)	Resistivity (×10^3^) (Ω cm)
As-grown	3.19 × 10^14^	10.13	1.93
0.01 M	9.83 × 10^14^	3.23	1.97
0.10 M	1.43 × 10^15^	2.29	1.88
0.15 M	1.12 × 10^15^	2.77	2.02
0.20 M	1.10 × 10^15^	3.01	1.91

## Data Availability

Not applicable.

## References

[1] Sarker E., Halder P., Seyedmahmoudian M., Jamei E., Horan B., Mekhilef S., Stojcevski A. (2021). Progress on the demand side management in smart grid and optimization approaches. Int. J. Energy Res..

[2] Wilson G.M., Al-Jassim M., Metzger W.K., Glunz S.W., Verlinden P., Xiong G., Mansfield L.M., Stanbery B.J., Zhu K., Yan Y. (2020). The 2020 photovoltaic technologies roadmap. J. Phys. D Appl. Phys..

[3] Sharma K., Poonam, Saini G.S.S., Tripathi S.K. (2018). A comparative study of transport properties of copper doped cadmium selenide thin films at two dopant concentrations. J. Mater. Sci. Mater. Electron..

[4] Green M., Dunlop E., Hohl-Ebinger J., Yoshita M., Kopidakis N., Hao X. (2021). Solar cell efficiency tables (version 57). Prog. Photovolt. Res. Appl..

[5] Li C., Wang F., Chen Y., Wu L., Zhang J., Li W., He X., Li B., Feng L. (2018). Characterization of sputtered CdSe thin films as the window layer for CdTe solar cells. Mater. Sci. Semicond. Process..

[6] Abdulkaleq S.Y., Al Taan L.M. (2021). Effect of Switching the Preparation Solution for CdSe Films by (CBD) Method on, Thickness, Optical and Structure Properties of the Films. Rafidain J. Sci..

[7] Song Z., Wang Y., Zhu Y., Bai P., Hu A., Gao Y. (2021). Targeted transfer of self-assembled CdSe nanoplatelet film onto WS2 flakes to construct hybrid heterostructures. J. Semicond..

[8] Ji Z., Song Z. (2023). Exciton radiative lifetime in CdSe quantum dots. J. Semicond..

[9] Patel S., Purohit A., Chander S., Kannan M., Dhaka M. (2018). An approach to MgCl_2_ activation on CdSe thin films for solar cells. Curr. Appl. Phys..

[10] Ojo A.A., Dharmadasa I.M. (2018). Electroplating of semiconductor materials for applications in large area electronics: A review. Coatings.

[11] Ibrahim M., Chelvanathan P., Mottakin M., Muhammad G., Miraz M.H., Akhtaruzzaman, Shahiduzzaman, Sobayel K., Kamal N. (2022). Effect of CuCl_2_ treatment on RF magnetron-sputtered CdSe thin films for potential photovoltaic usage. Jpn. J. Appl. Phys..

[12] Mahato S., Shakti N., Kar A. (2015). Annealing temperature dependent structural and optical properties of electrodeposited CdSe thin films. Mater. Sci. Semicond. Process..

[13] de la Cueva L., Lauwaet K., Otero R., Gallego J.M., Alonso C., Juarez B.H. (2014). Effect of chloride ligands on cdse nanocrystals by cyclic voltammetry and X-ray photoelectron spectroscopy. J. Phys. Chem. C.

[14] Munshi A.H., Kephart J.M., Abbas A., Danielson A., Gḗlinas G., Beaudry J.-N., Barth K.L., Walls J.M., Sampath W.S. (2018). Effect of CdCl_2_ passivation treatment on microstructure and performance of CdSeTe/CdTe thin-film photovoltaic devices. Sol. Energy Mater. Sol. Cells.

[15] Major J.D., Treharne R.E., Phillips L.J., Durose K. (2014). A low-cost non-toxic post-growth activation step for CdTe solar cells. Nature.

[16] Greenhalgh R.C., Abbas A., Munshi A.H., Shimpi T.M., Barth K.L., Sampath W.S., Bowers J.W., Walls J.M. Activation of thin film CdTe solar cells using a cadmium bromide treatment. Proceedings of the 2018 IEEE 7th World Conference on Photovoltaic Energy Conversion (WCPEC) (A Joint Conference of 45th IEEE PVSC, 28th PVSEC & 34th EU PVSEC).

[17] Leushina A.P., Danilov D.N., Zyablitseva E.V. (2008). Introduction of microdoses of germanium and indium dopants into the bulk and surface layers of semiconductor materials. Glas. Phys. Chem..

[18] Williams B.L., Major J.D., Bowen L., Keuning W., Creatore M., Durose K. (2015). A comparative study of the effects of nontoxic chloride treatments on CdTe solar cell microstructure and stoichiometry. Adv. Energy Mater..

[19] Macarie L., Simulescu V., Ilia G. (2019). Phosphonium-Based Ionic Liquids Used as Reagents or Catalysts. Chemistryselect.

[20] Harif M.N., Rahman K.S., Rosly H.N., Chelvanathan P., Doroody C., Misran H., Amin N. (2020). An approach to alternative post-deposition treatment in CdTe thin films for solar cell application. Superlattices Microstruct..

[21] Potlog T., Ghimpu L., Gashin P., Pudov A., Nagle T., Sites J. (2003). Influence of annealing in different chlorides on the photovoltaic parameters of CdS/CdTe solar cells. Sol. Energy Mater. Sol. Cells.

[22] Kim S., Song J.-Y., Kim D., Hong J., Cho I.J., Kim Y.H., Jeong J.-U., Yoon M.S., Ahn S.-J., Chung W.-K. (2021). Effect of novel double treatment on the properties of CdTe solar cells. Energy Rep..

[23] Cochran E.A., Woods K.N., Johnson D.W., Page C.J., Boettcher S.W. (2019). Unique chemistries of metal-nitrate precursors to form metal-oxide thin films from solution: Materials for electronic and energy applications. J. Mater. Chem. A.

[24] Santos J.L., Soares J.X., Rodrigues S.S.M., Ribeiro D.S. (2019). Semiconductor Quantum Dots in Chemical Analysis.

[25] Venkatachalam V., Ganapathy S., Perumal I., Priyadarshini N., Santhosh Jeferson Stanley J.S. (2023). The Size and Defect Controlled CdTe: In Colloidal Quantum Dots Via Varying the InCl3 Dopant Precursor Concentration in Aqueous Medium for Improving Solar Cell Performance. Inorg. Chem. Commun..

[26] Baines T., Zoppi G., Bowen L., Shalvey T.P., Mariotti S., Durose K., Major J.D. (2018). Incorporation of CdSe layers into CdTe thin film solar cells. Sol. Energy Mater. Sol. Cells.

[27] Kitazono K., Akashi R., Fujiwara K., Akita A., Naya S.I., Fujishima M., Tada H. (2017). Photocatalytic synthesis of CdS (core)–CdSe (shell) quantum dots with a heteroepitaxial junction on TiO_2_: Photoelectrochemical hydrogen generation from water. ChemPhysChem.

[28] Patel S., Himanshu, Chander S., Purohit A., Kannan M., Dhaka M. (2019). Understanding the physical properties of CdCl2 treated thin CdSe films for solar cell applications. Opt. Mater..

[29] Kumar V., Sandhu G.S., Sharma T.P., Hussain M. (2007). Growth and Characterization of Cd1− XZnXTe-Sintered Films. Res. Lett. Mater. Sci..

[30] Perna G., Capozzi V., Minafra A., Pallara M., Ambrico M. (2003). Effects of the indium doping on structural and optical properties of CdSe thin films deposited by laser ablation technique. Eur. Phys. J. B.

[31] Kotb H.M., Dabban M., Abdel-Latif A., Hafiz M. (2012). Annealing temperature dependence of the optical and structural properties of selenium-rich CdSe thin films. J. Alloys Compd..

[32] Sahebi R., Roknabadi M.R., Behdani M. (2020). Semi-transparent Schottky junction solar cell based on evaporated CdSe thin films: Influence of post-deposition air-annealing. Optik.

[33] Sarmah K., Sarma R., Das H.L. (2008). Structural characterization of thermally evaporated CdSe thin films. Chalcogenide Lett..

[34] Mahato S., Kar A.K. (2017). The effect of annealing on structural, optical and photosensitive properties of electrodeposited cadmium selenide thin films. J. Sci. Adv. Mater. Devices.

[35] Amarasinghe M., Sivananthan S., Metzger W.K., Colegrove E., Moutinho H., Albin D., Duenow J., Johnston S., Kephart J., Sampath W. (2018). Influence of CdTe deposition temperature and window thickness on CdTe grain size and lifetime after CdCl 2 recrystallization. IEEE J. Photovolt..

[36] Jamil N.Y., Burjus A.Y., Khalil H.M. (2018). The effect of ag doping on the structural, optical and electrical properties of CdSe thin films. Rafidain J. Sci..

[37] Patel S.L., Himanshu, Kaushalya, Chander S., Kannan M.D., Dhaka M.S. (2019). Toward CdCl_2_ activation on CdSe thin films for absorber layer applications. J. Mater. Sci. Mater. Electron..

[38] Sahebi R., Roknabadi M.R., Behdani M. (2020). Effect of Ag-doping on the structural, optical, electrical and photovoltaic properties of thermally evaporated Cadmium Selenide thin films. Mater. Res. Express.

[39] Chander S., Dhaka M. (2018). CdCl_2_ treatment concentration evolution of physical properties correlation with surface morphology of CdTe thin films for solar cells. Mater. Res. Bull..

[40] Punitha K., Sivakumar R., Sanjeeviraja C., Sathe V., Ganesan V. (2014). Physical properties of electron beam evaporated CdTe and CdTe: Cu thin films. J. Appl. Phys..

[41] Mahalingam T., Mariappan R., Dhanasekaran V., Mohan S.M., Ravi G., Chuc J.P. (2010). Characterization of electrodeposited indium doped CdSe thin films. JP Chu. Chalcogenide Lett..

[42] Mahato S., Kar A. (2015). Structural, optical and electrical properties of electrodeposited cadmium selenide thin films for applications in photodetector and photoelectrochemical cell. J. Electroanal. Chem..

[43] Stroyuk O., Raevskaya A., Gaponik N., Selyshchev O., Dzhagan V., Schulze S., Zahn D.R. (2018). Origin of the broadband photoluminescence of pristine and Cu+/Ag+-doped ultrasmall CdS and CdSe/CdS quantum dots. J. Phys. Chem. C.

[44] Smida A., Zaaboub Z., Mohamed N.B.H., Hassen M., Laatar F., Maaref H., Ezzaouia H. (2018). Photoluminescence behavior in the synthesized CdSe thin films deposited on ITO substrates. J. Lumin..

[45] Raut V.S., Lokhande C.D., Killedar V.V. (2017). Photoelectrochemical studies on electrodeposited indium doped CdSe thin films using aqueous bath. J. Electroanal. Chem..

[46] Hayashi T., Saeki R., Suzuki T., Fukaya M., Ema Y. (1990). Formation and properties of In-doped high-conductivity CdSe evaporated film. J. Appl. Phys..

[47] Takanoglu D., Yilmaz K., Ozcan Y., Karabulut O. (2015). Structural, electrical and optical properties of thermally evaporated cdse and in-doped cdse thin films. Chalcogenide Lett..

